# Biomarkers in Endurance Exercise: Individualized Regulation and Predictive Value

**DOI:** 10.1155/2023/6614990

**Published:** 2023-12-14

**Authors:** Sebastian Hacker, Johannes Keck, Thomas Reichel, Klaus Eder, Robert Ringseis, Karsten Krüger, Britta Krüger

**Affiliations:** ^1^Department of Exercise Physiology and Sports Therapy, Institute of Sports Science, Justus-Liebig-University Giessen, Giessen, Germany; ^2^Nemolab, Institute of Sports Science, Justus-Liebig-University Giessen, Giessen, Germany; ^3^Institute of Animal Nutrition and Nutrition Physiology, Justus-Liebig-University, Giessen, Germany

## Abstract

The high interindividual variability of exercise response complicates the efficient use of blood-based biomarkers in sports. To address this problem, a useful algorithm to characterize the individual regulation and predictive value of different candidate markers will be developed. Forty-nine participants completed two identical exercise trials. Blood samples were collected before, immediately after, 3 hours after, and 24 hours after completion of exercise. Plasma concentrations of interleukin (IL-) 1RA, IL-6, IL-8, IL-10, IL-15, creatine kinase (CK), cortisol, c-reactive protein (CRP), lactate dehydrogenase (LDH), and thiobarbituric acid reactive substances (TBARS) were measured. Individualized regulation was analyzed using k-means clustering and a Group Assignment Quality (GAQ) score. Regression trees with a bootstrapped-aggregated approach were used to assess the predictive qualities of the markers. For most of the markers studied, a distinction can be made between individuals who show a stronger or weaker response to a particular endurance training program. The regulation of IL-6, IL-8, IL-10, and CK exhibited a high degree of stability within the individuals. Regarding the predictive power of the markers, for all dependent variables, the most accurate predictions were obtained for cortisol and IL-8 based on the baseline value. For CK, a good prediction of recovery of maximal strength and subjective feeling of exhaustion can be made. For IL-1RA and TBARS, especially their reregulation can be predicted if the baseline level is known. Focusing individual variations in biomarker responses, our results suggest the combined use of IL-6, IL-8, IL-10, and CK for the personalized management of stress and recovery cycles following endurance exercise.

## 1. Introduction

The use of blood-based biomarkers to control exercise and training is becoming gradually important since the demands and density of competitions are increasing. In addition, the scientific knowledge for the identification of candidate markers via OMICS methods is advancing, and more new markers are being investigated, especially in the view of measurement methods becoming more precise, less expensive, and more mobile [1, 2].

A biomarker can be one of the numerous molecules secreted during and after exercise and induce a physiological response resulting in a relative disturbance of homeostasis in various organs and tissues [2]. This disturbance of homeostasis depends on the intensity and duration and, thus, on the relative stress of the exercise bout. The quantity of released molecules thereby reflects the level of stress of the affected systems. After exercise cessation, these molecules are regulated back to baseline, reflecting the return to homeostasis and further the regeneration or adaptation process of the corresponding physiological system [1]. Ideally, the potential biomarker can then be used to identify which system was disturbed and when in time it is regenerated. This would be of particular interest as these markers might be used for the differential and individual control of exercise load and regeneration.

Possible candidate markers include, above all, immunological markers such as interleukin (IL-) 1RA, IL-6, or IL-8, since every acute bout of exercise is accompanied by an immune reaction [3]. Other molecules reflect the exercise-induced metabolic stress, such as cortisol, or the disruption of tissue integrity, resulting in the release of intracellular enzymes, such as creatine kinase (CK) [4] or lactate dehydrogenase (LDH) [5]. A recent study by Reichel et al. explored the reliability and the exercise-related kinetics of a large number of these candidate markers [1]. Their analysis identified, for example, thiobarbituric acid reactive substances (TBARS), LDH, and IL-1RA, to be suitable markers that might be helpful for monitoring athletes' load management.

One of the central goals of modern elite sports is to individualize the control of exercise load and training, tailoring it to the individual athlete's responses [6]. However, the traditional scientific view often neglects individual differences in the exercise-dependent response and regulation of the released molecules. Thus, individual differences were often only reflected in the depicted variance and were partly ignored, resulting in little knowledge about the individual regulation of blood-based biomarkers. For some markers, however, such as CK, it has already been shown that there are individuals with a stronger or weaker response due to different genetic variants, which lead to a differing stability of the muscular sarcolemma [7]. A detailed knowledge of the individual response pattern would then allow a better interpretation of the respective measurement of the biomarker. Accordingly, the degree to which various biomarkers are subject to an individual regulatory profile becomes a key research question, which must subsequently be considered in the interpretation of the measurements in the context of exercise monitoring [8]. Another unresolved issue is to what extent the measurement of these markers can be used to make predictions regarding its reregulation, as well as regarding subjective and objective recovery. This predictive value of biomarkers is one of the central requirements for a marker to be used in practice [8]. Predictability of the regulation means that the knowledge of the individual regulation can also be used to predict how the athlete would react to a given load regarding this biomarker. This could be important for coaches and athletes at many points of training management, for example, in the preparation of important competitions, or during tournaments with several subsequent single competitions.

On this background, the aim of our study was to determine whether different blood-based biomarkers show an individual response pattern after acute bouts of endurance exercise. We addressed the question whether this response pattern shows stability for each individual over two days of measurement what might open up the possibility of using the respective marker as a criterion for physical training state, strain, fatigue, and recovery. Furthermore, we looked at the predictive capacity of each biomarker to predict its reregulation, the recovery of maximal strength, and the subjective feeling of recovery. Specifically, we quantified the response kinetics of 10 hormones, enzymes, cytokines, and correlates of oxidative stress after two identical exhausting 60 min running trials. We then analyzed the individual response kinetic of the measured blood-based biomarkers by using k-means clustering to group similar individual responses to the exercise stimulus for each day and determined the characteristics of the groups. We further quantified whether an individual is assigned to the same group on each day and the response pattern of each biomarker is a characteristic of each individual. In a last step, we investigated the predictive quality of each biomarker to predict the value 24 hours after exercise exposure as well as the recovery of maximal strength and the subjective feeling of recovery. Therefore, we applied regression trees, where we additionally determined the relative importance of each predictor.

## 2. Methods

### 2.1. Subjects

The study is a machine learning reanalysis of a subgroup of a larger collective whose data have already been published in other analyses and contexts [1]. A total of 49 male and female subjects (male: 25, female 24) with different training status participated in the study. Personal characteristics and anthropometric data are shown in Table 1. To ensure that all subjects were physically healthy and able to participate in sporting activities, they were medically screened. Exclusion criteria consisted of smoking, pregnancy, mothers in the lactation period, cardiovascular diseases, acute infections, musculoskeletal injuries, acute symptomatic respiratory deficits, and chronic diseases. All procedures were approved by the local Ethics Committee of the Department of Psychology and Sports Science of the Justus-Liebig-University Giessen and adhered to the Declaration of Helsinki. All participants provided written informed consent prior to participating.

### 2.2. Experimental Approach

#### 2.2.1. Preliminary Testing

In a first step, we tested the endurance capacity of each participant to monitor the kinetics of various markers during two following identical strenuous exercise trials under controlled conditions. Endurance capacity was determined by a continuous progressive exercise field test using lactate diagnostics. On a 200 m running track, subjects started at 6 km/h and increased their running speed by 2 km/h every three minutes until subjective exhaustion (using the BORG scale). Prior to the field test, between all three-minute stages with a break of 30 s and immediately after exhaustion, 20 *µ*L of capillary blood was taken from the earlobe with an end-to-end glass capillary. The heart rate (HR) was continuously tracked using HR monitors (Polar FT1, Polar Electro Oy, Finland). Blood lactate values were subsequently analyzed using enzymatic-amperometrical detection (Bosen S-Line Plus, EKF-Diagnostics Sales GmbH, Magdeburg, Germany). HR and blood lactate values were used to evaluate the individual anaerobic threshold (IAT) using the Ergonizer Software for medical application (Ergonizer Software 4.9.4, Freiburg, Germany). The IAT was used to determine the individual running intensity during the following two strenuous exercise trials. Calculation of IAT was performed by adding the constant value of 1.5 mmol/L to lactate concentration at the individual's lactate threshold. To investigate the maximum voluntary contraction (MVC) of the knee flexors and extensors, an isometric strength test utilizing the m3diagnos dynamometer (Schnell, Peutenhausen, Germany) was conducted as described elsewhere [1].

#### 2.2.2. Testing Days of Strenuous Exercise Trials

**Figure 1:**
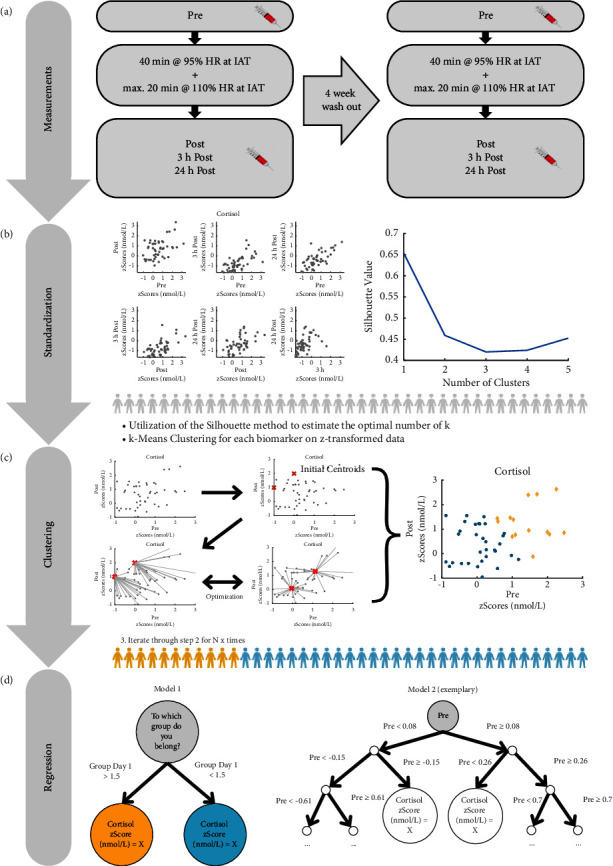
Data sampling and data analysis. (a) Time schedule of the experimental procedure. (b) *z*-standardization was performed for each biomarker before (c) k-means clustering and (d) regression were calculated. Predictions were based on two different models. A binary group model (model 1) or a bag of regression trees based on the prevalue (model 2).

Approximately one week after preliminary testing, the first of two strenuous exercise trials took place. Both testing days (TDs) started between 8:00 and 9:00 am for each subject. Prior to the TDs, subjects were instructed on several standardized conditions to which they had to comply. From four days before the TDs, subjects were not allowed to take part in any exhausting physical activity; only regenerative training was acceptable. Furthermore, it was forbidden to consume alcohol the day before. A nutrition protocol had to be drawn up, which included all consumed drinks and meals one day prior to TD1 as well as breakfast on the TD. The protocol served as a guideline for food intake prior to the second testing day (TD2) to ensure standardized conditions. At the respective testing day, subjects had to fill out a questionnaire concerning their regular physical activity and their usual nutrition. All participants did not change their regularities in nutrition as well as in physical activity in between the exercise trials. Furthermore, all female subjects documented their menstrual cycle. These questionnaires were issued to document large deviations in these habits and to exclude possible changes in physical performance between TD1 and TD2. The exhaustive physical activity on both TDs consisted of two identical 60-min continuous endurance running field tests (RFTs), intermitted by a recovery period of approximately four weeks. Highly standardized conditions were created for both testing days. The exercise protocol consisted of 40 min running at an intensity corresponding to 95% of HR at IAT, followed by 20 min at 110% to ensure exhaustion and the same relative exercise intensity for all subjects. All participants completed both RFTs at the same duration at the respective HR. Blood samples were collected before, immediately after, 3 h, and 24 h after each exercise test. The complete study design is presented in Figure 1(a).

#### 2.2.3. Blood-Based Biomarkers

Venous blood samples were collected at four points in time on each testing day (prior to exercise = t1; immediately after exercise = t2; 3 hours after exercise = t3; 24 hours after exercise = t24) in vacutainers. Plasma vacutainers were anticoagulated with EDTA. The vacutainers were centrifuged at 2,500 × g for 10 min at 4°C immediately after sampling, while serum samples had clotted for 30 min before centrifugation. Samples were separated into aliquots and stored in Eppendorf tubes at −80°C until analysis. The measurement methodology and analytical sensitivities of the assays have already been described by Reichel et al. [1]. Briefly, IL-1RA, IL-6, IL-8, IL-10, and IL-15 were determined by high-sensitivity ELISA (Quantikine ELISA Kits: R&D Systems, MVZ, Koblenz, Germany). Enzymes, CK and LDH, as well as the plasma protein CRP, were analyzed by ELISA using a Cobas 8,000 immunoassay system (Roche Diagnostics). Levels of cortisol and IL-6 were measured by using an Advia Centaur XPT immunoassay system (Siemens MVZ, Koblenz, Germany). Plasma concentration of TBARS, a metabolite of lipid peroxidation, was determined spectrofluorimetrically (Fluorescence Spectrometer LS55, PerkinElmer, Rodgau, Germany).

### 2.3. Statistics

As a first step, we standardized the values of all 10 biomarkers via *z*-transformation for both days to make their values directly comparable.

#### 2.3.1. Classification: k-Means Clustering

In the next step, we performed k-means clustering. We used this unsupervised machine learning approach to cluster individuals that are characterized by similar response patterns under the strenuous exercise performance on TD1 and TD2. Using the k-means cluster method requires a given number of clusters. To extract this parameter from our data, we calculated the silhouette value for different group sizes. We set a cut off at six groups to prevent overfitting while simultaneously uncovering practical and relevant differences. The silhouette value gives us an approximation of the optimum group size. The closer the value is to 1, the more similar the groups are within their clusters and the less similar they are to the other clusters (Figure 1(b)). For most biomarkers, *k* = 2 was found to be the optimal number of possible clusters based on the data of TD1. We therefore decided to use *k* = 2 for all biomarkers on both days to ensure comparability across markers and days. Next, we performed 10.000 iterations with *k* = 2. Per iteration, 5 different initial centroids were used, and the one with the best arrangement was chosen for each iteration.

#### 2.3.2. Determining the Group Assignment Quality (GAQ) Score

To quantify whether an individual is assigned to the same cluster and, therefore, has a stable group membership on both days, we defined a Group Assignment Quality (GAQ) score. We calculated the standard deviation over both days (0 = same group on both days over all iterations; 0.5 = one group on one day and the other group on the other day), as the k-means approach is susceptible to variance in the dataset. This measure defines the variation across all iterations and depicts the stability of the assignment of each subject to a group. Last, we have subtracted the standard deviation over both days and iterations from 1 to make it easier to interpret. High values correspond to a high degree of certainty in the group assignment and vice versa:

(1) $$\begin{array}{c} \mathrm{GAQ}=1-\mathrm{std}\;\left[\mathrm{group}\;\mathrm{assingment}\;\mathrm{day}\;1,\mathrm{group}\;\mathrm{assignment}\;day\;2\right]. \end{array}$$

**Figure 2:**
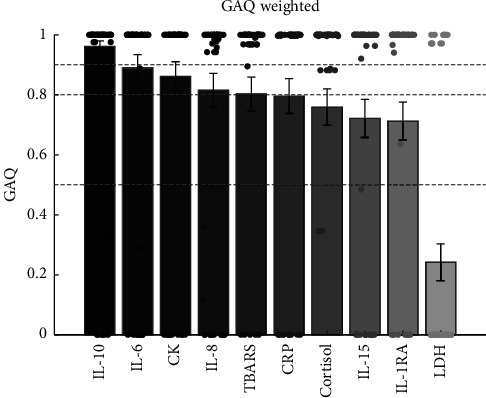
Group Assignment Quality (GAQ) score. For each biomarker, the GAQ and SEM were calculated and then sorted in descending order. Individuals are indicated by dots. High values correspond to a high degree of certainty in the group assignment and vice versa.

In order to give more weight to subjects who changed group memberships across the two intervention days, we further multiplied the individual standard deviations per subject by a value between 0 (same group across all iterations on both days) and 2 (one group on one day and another on another day). We have applied this weighting because we want to represent group changes more strongly as this event is opposed to group stability. This results in a GAQ value of 0 for all uncertain assignments over both days. The closer the GAQ tends to 1, the more certain we are about the group assignment. For example, if a marker has a GAQ of 0.8, this means that 9 of 49 participants have changed the group across the two days, whereas a GAQ of 0.96 means that none of the participants changed the group (GAQ weighted, Figure 2).

#### 2.3.3. Group Characterization

To describe the key features and differences (pre- and post-exercise level, the responsiveness of the group members, the ability to recover, etc.) of the determined groups for each biomarker, we calculated the mean baseline level, the mean postexercise level, the mean initial increase after the endurance exercise, and the mean performance capacity as well as the recovery and reregulation capacity (i.e., the difference of t24  minus t1 for TD1 and t24  minus t1 for TD2, respectively) for each group. We further tested whether the determined groups revealed significant differences with respect to these features via independent *t*-tests. In case of distribution violations, we used the Mann–Whitney *U*-Test. Our significance level was set at *p* ≤ 0.05.

#### 2.3.4. Prediction: Regression Trees

To explore the potential of each biomarker to predict aspects of reregulation as well as objective and subjective measures of performance and recovery, regression tree classifiers were trained using MATLAB Statistics and Machine Learning Toolbox (version 12.0) with a bootstrapped-aggregated approach. Prediction of the biomarker concentration at 24 h post exercise and the subjective feeling of exhaustion and recovery (BORG scale) as well as the recovery of the maximum isometric quadriceps flexion (t24-t1) was performed using different models with varying features and increasing complexity. Model 1 included only the grouping variable of the cluster analysis, model 2 used t1 (i.e., the baseline value), and model 3 consisted of the combination of group assignment and t1 of TD1. Afterwards, we compared the performances of the different models and examined which feature is the most suitable for prediction of the aforementioned outcomes. It must be emphasized that training and validation were based on the data delivered from TD1, while model performance was evaluated with data delivered from TD1 and TD2 as test datasets by computing root mean squared errors (RMSE). The decision to also use data from TD2 as another test dataset is because it can be considered completely independent of TD1. If a good prediction is also possible for this independent dataset, this underpins the stability of a marker.

#### 2.3.5. Determining the Quality of Candidate Markers

In a last step, we ranked the GAQ values as well as the summed RMSEs across all predictions to identify the most suitable markers depending on their performance in both analyses by determining the average rank for each marker.

The data analysis procedure is shown again in Figure 1(b)–(d). All statistical analyses were carried out using MATLAB version R2020b Update 4 (the MathWorks Inc., Natick, Massachusetts, USA) and JASP version 0.17.1 (JASP Team, University of Amsterdam, Amsterdam, Netherlands). Figures were created with MATLAB and Canva for Teams version 1.78.0 (Canva Pty Ltd, Sydney, Australia).

## 3. Results

### 3.1. Cluster Analysis

**Figure 3:**
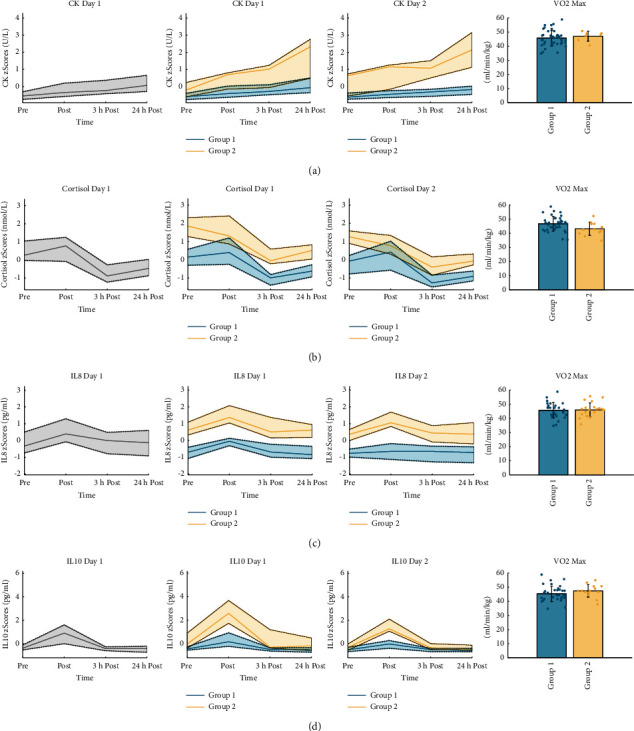
Grouping results based on k-means clustering. Depicted are the median and the 25% quantile for the total group (left column) in grey and for both groups after clustering for day 1 (second column) and day 2 (third column) as well as VO_2_max for the corresponding groups. (a) CK kinetics for the whole group and for the identified subgroups for both testing days, as well as the VO_2_max for the subgroups. (b) Cortisol for the whole group and for the identified subgroups for both testing days, as well as the VO_2_max for the subgroups. (c) IL-8 kinetics for the whole group and for the identified subgroups for both testing days, as well as the VO_2_max for the subgroups. (d) IL-10 kinetics for the whole group and for the identified subgroups for both testing days, as well as the VO_2_max for the subgroups.

For CK, cluster 1 consists of 42 subjects and cluster 2 consists of 7 subjects (Figure 3(a)). Significant differences were found with respect to the CK level prior to exercise (*M*1 = −0.56, SD1 = 0.26; *M*2 = 0.04, SD2 = 0.43; *p*  < 0.001), the postexercise level (*M*1 = −0.34, SD1 = 0.35; *M*2 = 0.8, SD2 = 0.39; *p*  < 0.001), the initial exercise response (*M*1 = 0.22, SD1 = 0.12; *M*2 = 0.76, SD2 = 0.47; *p*  < 0.001), and to the recovery period (*M*1 = 0.6, SD1 = 0.46; *M*2 = 2.73, SD2 = 1.12; *p* < 0.001). Group 2 showed a higher baseline and postexercise blood concentration and a steeper increase and decrease in CK concentration than group 1. The GAQ for CK was 0.86, which means a rather stable assignment for each individual to a given cluster reflecting that the respective CK response seems to be a characteristic response for the individual under strenuous endurance exercise (Figure 2).

For LDH, two clusters were observed, whereby cluster 1 consists of 26 subjects and cluster 2 consists of 23 subjects. Significant differences were found with respect to the LDH level prior to exercise (*M*1 = −0.3, SD1 = 0.62; *M*2 = −1.24, SD2 = 0.41; *p*  < 0.001), the postexercise level (*M*1 = 1.31, SD1 = 0.63; *M*2 = −0.11, SD2 = 0.46; *p*  < 0.001), the training status (VO_2_max equivalent) (*M*1 = 48.04, SD1 = 4.47; *M*2 = 43.49, SD2 = 5.08; *p* = 0.002), and to the initial exercise response of both clusters (*M*1 = 1.62, SD1 = 0.47; *M*2 = 1.13, SD2 = 0.46; *p*  < 0.001). Group 1 showed a higher pre- and post-exercise level of LDH as well as a steeper increase during exercise. Furthermore, group 1 showed a higher VO_2_max. The GAQ of LDH, however, was rather poor at 0.24 (Figure 2), reflecting a rather low stability of the group assignment across the two testing days.

The k-means clustering of the cortisol response of 49 individuals resulted in two clusters. Cluster 1 consists of 37 subjects, and cluster 2 consists of 12 subjects. Both clusters were well separated from each other as illustrated in Figure 3(b). By determining the key features of each cluster, we found significant differences with respect to the cortisol level prior to exercise (*M*1 = 0.13, SD1 = 0.61; *M*2 = 1.84, SD2 = 0.68; *p*  < 0.001), the postexercise level (*M*1 = 0.41, SD1 = 0.73; *M*2 = 1.6, SD2 = 0.88; *p*  < 0.001), the training status (VO_2_max equivalent) (*M*1 = 46.8, SD1 = 5.15; *M*2 = 43.15, SD2 = 4.71; *p* = 0.035), and to the recovery period of both clusters (*M*1 = −0.74, SD1 = 0.7; *M*2 = −1.4, SD2 = 0.53; *p* < 0.01). Group 1 is characterized by a lower pre- and post-exercise level, a shorter recovery period, and a higher VO_2_max. The GAQ for cortisol is 0.76 (Figure 2), reflecting a quite moderate stability for assigning an individual to the same group on both testing days.

TBARS response kinetic resulted in two clusters, which were relatively evenly distributed. Cluster 1 consists of 27 subjects, and cluster 2 consists of 21 subjects. Significant differences between groups were found for pre-exercise levels (*M*1 = −0.74, SD1 = 0.47; *M*2 = 0.61, SD2 = 0.62; *p*  < 0.001) and for postexercise levels (*M*1 = −0.18, SD1 = 0.59; *M*2 = 1.37, SD2 = 0.72; *p*  < 0.001). Group 1 revealed lower pre- and post-exercise levels. For TBARS, a GAQ of 0.8 was determined.

The k-means clustering of the CRP response kinetic resulted in two clusters, whereby cluster 1 consists of 41 subjects and cluster 2 consists of 8 subjects. Both clusters were well separated from each other. We found significant differences with respect to the CRP level prior to exercise (*M*1 = −0.51, SD1 = 0.3; *M*2 = 1.68, SD2 = 0.75; *p*  < 0.001) and the postexercise level (*M*1 = −0.51, SD1 = 0.31; *M*2 = 1.84, SD2 = 0.82; *p*  < 0.001), whereas group 1 revealed lower prelevels and postlevels. The GAQ was 0.8 (Figure 2).

For IL-1RA, 31 subjects formed cluster 1 and 17 subjects formed cluster 2. Both clusters were well separated from each other. Significant differences were found with respect to the IL1RA level postexercise (*M*1 = −0.21, SD1 = 0.92; *M*2 = 0.33, SD2 = 0.86; *p*  < 0.01) and the initial exercise response (*M*1 = 0.34, SD1 = 0.38; *M*2 = 0.71, SD2 = 0.68; p = 0.017), whereas group 1 revealed lower prelevels and postlevels of IL1RA. GAQ for IL1RA was 0.71 (Figure 2).

For the IL-6 response, two clusters were identified, whereby cluster 1 consisted of 41 subjects and cluster 2 consisted of 8 subjects. Significant differences were found with respect to the IL-6 level prior to exercise (*M*1 = −0.71, SD1 = 0.34; *M*2 = 0.65, SD2 = 0.76; *p* < 0.001) and the postexercise level (*M*1 = 0.84, SD1 = 0.97; *M*2 = 2.1, SD2 = 1.77; *p* = 0.035). Group 1 revealed lower prelevels and postlevels of IL-6. The GAQ was 0.89 (Figure 2).

For IL-8, two clusters were found. Cluster 1 consisted of 28 subjects, and cluster 2 consisted of 21 subjects (Figure 3(c)). We found significant differences with respect to the IL-8 level before exercise (*M*1 = −0.68, SD1 = 0.47; *M*2 = 0.68, SD2 = 0.65; *p* < 0.001) and the postexercise level (*M*1 = -0.08, SD1 = 0.45; *M*2 = 1.56, SD2 = 0.81; *p* < 0.001). Group 1 revealed lower prelevels and postlevels. The GAQ was 0.82 (Figure 2).

IL-10 analysis resulted in two clusters of 35 and 14 subjects, respectively (Figure 3(d)). Significant differences were found with respect to the IL-10 level prior to exercise (*M*1 = −0.38, SD1 = 0.41; *M*2 = 0.42, SD2 = 1.23; *p* = 0.013), the postexercise level (*M*1 = 0.32, SD1 = 0.67; *M*2 = 2.81, SD2 = 1.35; *p*  < 0.001), and the initial exercise response (*M*1 = 0.7, SD1 = 0.76; *M*2 = 2.39, SD2 = 1.94; *p*  < 0.001). Group 1 revealed a lower prelevel and postlevel as well as a flatter increase. The GAQ was very high with 0.96, reflecting a high group stability (Figure 2).

Similarly, the IL-15 response resulted in two clusters. Cluster 1 included 26 subjects, while cluster 2 included 23 subjects. Significant differences were found with respect to the IL-15 level prior to exercise (*M*1 = −0.76, SD1 = 0.36; *M*2 = 0.64, SD2 = 0.71; *p* < 0.001) and the postexercise level (*M*1 = −0.02, SD1 = 0.47; *M*2 = 1.52, SD2 = 1.09; *p* < 0.001). Group 1 revealed a lower prelevel and postlevel. The GAQ was 0.72 (Figure 2).

### 3.2. Predictive Values of Blood-Based Biomarkers

By calculating regression trees, we analyzed the potential of each marker to predict its reregulation as well as objective and subjective measures of performance and recovery. The results of the best-performing model for each biomarker at TD1 and TD2 are presented in Table 2. The complete results table with all calculated models can be found in Supplement 1 (S1). For all three output variables (biomarker concentration at 24 h, subjective exercise response (BORG scale), and the difference in isometric quadriceps flexion (24 h post minus pre)), the prediction is best achieved via model 2, which uses the baseline level (t1) of the corresponding marker for prediction. This applies to all biomarkers.

The most accurate predictions based on the baseline level are obtained for all dependent variables for cortisol and IL-8. For CK, a good prediction of recovery of maximum strength and subjective feeling of exhaustion can be made. For IL-1RA and TBARS, especially their reregulation can be predicted when knowing the baseline level.

#### 3.2.1. Possible Candidate Markers

By creating two rankings (for prediction performances and for the calculated GAQ) and averaging the ranks for each marker, we determined the best-performing markers over the two analyses. After this, CK, cortisol, IL-8, and IL-10 appear to be the most suitable biomarkers.

## 4. Discussion

A central goal of modern elite sports is to individualize the management of exercise load and training [6]. While every athlete initially experiences subjective exhaustion in the context of load and recovery in terms of an internal load, also various body systems, such as muscular integrity, metabolism, or the immune system, are brought out of homeostasis and reregulate during recovery. This leaves molecular traces in the blood which can be used as objective markers for the differential quantification of these processes. Many studies point to a rather unstable interindividual regulation of these markers by high standard deviations, hindering many studies from yielding clear results in the application of various molecules as biomarkers [9]. The present study elucidated that various blood biomarkers exhibit differential response patterns after acute bouts of endurance exercise. For most of the markers studied, a distinction can be made between individuals who show a stronger responseand others who show a weaker response to a similar endurance exercise program (e.g., IL-10 and CK). In addition, our data revealed that individuals also differ in terms of basal and postexercise levels of some markers (e.g., IL-8 and cortisol). These differences in baseline and response behavior reveal the necessity of a tailored treatment of each physiological system from which the biomarker originates. The present study further provided evidence that the response pattern for IL-6, IL-8, IL-10, and CK exhibited a high degree of stability within the investigated individuals, implicating a high intraindividual reliability for the given markers. Our results further show that, depending on the candidate marker, exercise and recovery cycles can be predicted with reasonable precision for specific markers. The most accurate predictions are obtained for cortisol, the cytokines IL-8 and IL-1RA, and CK. Given the baseline level, subjective and objective recovery as well as reregulation can be best predicted for cortisol and IL-8 compared to the other markers. For CK, a good prediction of recovery of maximum strength and subjective feeling of exhaustion can be made. For IL-1RA, especially its reregulation can be predicted when knowing the baseline level. When merging the results of all conducted analyses, CK, IL-8, IL-10, and cortisol appear to be the best-performing biomarkers in the context of the present study. In the next paragraphs, we will discuss these possible candidates and their suitability in more detail.

### 4.1. Cortisol as a Biomarker for Exercise Control?

Cortisol is a glucocorticoid hormone secreted by the adrenal cortex in response to physical, psychological, or physiological stressors [10, 11]. Exercise is one such stressor that has been shown to significantly alter the circulating amounts of cortisol in the human body [12, 13]. This is due to the fact that exercise causes the activation of the hypothalamus resulting in the release of corticotropin-releasing hormone (CRH), which then stimulates the anterior pituitary to secrete adrenocorticotropic hormone (ACTH) followed by the release of cortisol from the adrenal cortex [14]. The present results revealed two groups differing regarding their pre- and post-exercise level, by reregulation to baseline as well as with regard to their VO_2_max, whereby group 1 exhibited lower pre- and post-exercise levels, a shorter recovery period, and a higher VO_2_max. In this regard, a study by Lucertini et al. showed that, in healthy elderly men, higher cardiorespiratory fitness levels are associated with a lower diurnal cortisol output and with minor effects on the cortisol response to acute mental stress [15]. Among the subjects who responded to mental stress, the amplitude of cortisol response and the steepness of recovery decline displayed an increasing trend in the high fit subjects. These as well as our data pin to the notion that higher fitness might lead to reduced cortisol levels and a reduced responsiveness of the hypothalamic-pituitary-adrenal axis (HPA), as well as to a shorter recovery period. It is possible that the cause of the influence of training status on cortisol levels is that while acute exercise stimulates the HPA axis, regular training induces an adaptation of HPA axis activity to repeated exercise. Thus, particularly intense physical training might lead to adaptive changes in basal HPA function, including a phase shift and increased pituitary ACTH secretion, but also blunting of the adrenal cortisol response. The fact that cortisol levels seem to reflect exercise-induced changes is interesting in terms of its potential use as a biomarker [11].

It is noteworthy that cortisol is one of the tested markers for which subjective and objective recovery as well as reregulation could be best predicted only by knowing its baseline level. Thus, cortisol as a molecule reflecting the (exercise-induced metabolic) stress-level appears to be very promising for predicting recovery cycles. However, the GAQ for cortisol is 0.76 (Figure 2), reflecting only a moderate stability over the two days and some interchanges for assigning an individual to the same group on both testing days. Hence, it must be stated that cortisol has a clear circadian rhythm with flatter and wavier diurnal cortisol curves depending on many behavioral and health factors. This characteristic must be considered as a confounding factor that makes cortisol difficult to use as a biomarker without control measurements outside of sports [16], including, for example, sleep-wake cycles, diet, and the daytime.

### 4.2. IL-8 as a Biomarker for Exercise Control?

IL-8 is an important chemotactic factor involved in neutrophil granulocyte recruitment and activation. It can be secreted by many structural and immune cells, including macrophages, bronchial epithelial cells, and muscle cells, which also classifies it as a myokine. IL-8 increases in response to the intensity and duration of exercise, and the increase seems to be somewhat flatter but more persistent than for IL-6 [17]. This is because IL-8 is probably released mainly in the muscle to act paracrine and autocrine as an angiogenic factor in human microvascular endothelial cells [18]. As an inflammatory mediator, it seems to be quite clearly assigned to the dimension of the exercise-induced immune response. Since it is affected in many disease states, such as asthma, its stability as a biomarker is to be expected especially in healthy individuals [19]. Our results point to two groups that differ in terms of their initial level as well as their level after exhausting exercise. Thus, there are individuals with a significantly higher basal level of IL-8. The assignment to the groups is quite stable. Different basal levels of IL-8 could be due to present, even small, sources of inflammation, such as periodontitis, or could be due to genetic factors, such as polymorphisms in genes related to IL-8 and CXCR2 [20]. However, these variables were not assessed in this study, and we can only speculate on it.

The present results also show that the basal levels of IL-8 are very well suited for predicting reregulation, the subjective exhaustion, and the recovery of muscle function 24 hours after exercise. This is particularly significant for practical application as a biomarker in sports, as it allows effective planning of a regeneration cycle and thus the start of the next training session in a reasonable and adjusted period. The use in a competition or a match can also be planned based on an objective parameter against the background of sufficient recovery. Physiologically, we would interpret the role of IL-8 here as being directly involved in the recovery process as an inflammatory parameter, particularly in the phase in which inflammatory tissue repair takes place, which then transitions into a reparative remodeling process.

### 4.3. Creatine Kinase as a Biomarker for Exercise Control?

CK is an enzyme that is found in higher concentrations in muscle cells and only slightly concentrated in plasma. The appearance of CK in blood has been generally considered to be an indirect marker of muscle damage, particularly for diagnosis of medical conditions such as myocardial infarction, muscular dystrophy, and cerebral diseases [21]. After muscular exercise, plasma CK levels increase, indicating a loss of integrity of the sarcolemma. CK is one of the few blood parameters that are more appropriately used in competitive sports [4]. Here, CK is used to diagnose muscular recovery with test strips and point-of-care analysis. However, there is controversy in the literature concerning its validity in reflecting muscle damage as a consequence of the level and intensity of physical exercise. Nonmodifiable factors, for example, ethnicity, age, and gender, can also affect enzyme tissue activity and subsequent CK serum levels [21]. The present results revealed two groups that differ with respect to their CK level prior to exercise, the postexercise level, and the initial exercise response as well as to the recovery period. Group 2 showed a higher baseline and postexercise blood concentration and a steeper increase and decrease in CK concentration. Thus, individuals in this group react much more sensitively and intensively to the exercise stressor and that their recovery times are also longer. The GAQ for CK was 0.86, which means that the respective CK response seems to be a characteristic response for the individual under strenuous endurance exercise. This is supported by data revealing that there are high responders regarding CK, who develop very high values after exercise, and low responders, with only a very flat response what seems to be genetically based and related to the stability of the muscle architecture [22].

Our data further showed that there is a clear relationship to muscular recovery and to the subjective sensation of exertion as both can be predicted when knowing the baseline level prior to exercise. This is consistent with the literature, which has also already linked muscle pain to the CK response [23]. Consequently, when using CK as a biomarker, it is critical to determine the athlete's baseline level and gather knowledge about the athlete's specific group assignment to ensure a proper individualized evaluation.

### 4.4. Il-10 as a Biomarker for Exercise Control?

IL-10 is expressed by cells of many leukocyte subpopulations including macrophages, natural killer cells, and T cells. It is a rather immunoregulatory and anti-inflammatory cytokine that has a strong downregulatory effect on the secretion of proinflammatory cytokines such as IL-1, IL-1*β*, and TNF-*α*. The magnitude of the increase in the concentration of IL-10 seems to be mainly dependent on the release of IL-6 and the exercise duration. It is not yet fully understood what triggers the increase in IL-6 during exercise. There are indications of a progressive depletion of glycogen stores as well as leaky gut phenomena during prolonged exercise, which initially induce a pro-inflammatory and then an anti-inflammatory counterreaction [24]. The degree to which group differences are due to genetic factors around IL-6 polymorphisms remains rather speculative [25].

IL-10 analysis resulted in two clusters of 35 and 14 subjects, respectively, whereby group 2 revealed a higher pre- and post-exercise level as well as a steeper increase speaking to the notion that that there are IL-10-high- and low responders to exercise. The GAQ was very high with 0.96, reflecting that none of the participants changed groups over the two testing days. However, IL-10 does not perform as well as the other markers in predicting recovery. IL-10 triggers changes in macrophage phenotypes that promote muscle growth and regeneration. Due to its secondary release to an inflammatory stimulus, IL-10 might be less directly related to recovery than the other markers [26].

### 4.5. Should We Use a Panel of Different Biomarkers?

Despite the remarkable stability and predictive value of single biomarkers, it would be worth considering a combination of biomarkers in the form of a panel for the diagnosis of exercise response and recovery processes. The risk in the use of individual biomarkers is always a certain susceptibility to error as well as the limited informative value for individual physiological systems [27]. A panel of markers identified here as particularly stable and predictive would add particular value in their use as biomarkers to diagnose exercise recovery cycles. A combined analysis of cortisol, IL-6, IL-8, IL-10, and CK can be very helpful in giving information about the homeostasis of different systems. While interleukins are markers related to inflammation, cortisol is a stress marker related to metabolism and CK is a marker related to muscle damage. Accordingly, by using this marker panel, a clearer and more holistic picture can be obtained of athletic stress regarding different physiological systems. The differentiated regulation of the markers can then provide valuable information on the recovery process and possibly be used for differentiated and personalized recovery management [28].

### 4.6. Methodological Considerations

We scheduled two exercise sessions with a four-week interval between them to prevent any carryover effects during this period. The repeated bout effect is mainly shown after eccentric exercises [29]. For the exercise unit, we opted for a moderate intensity but exhaustive regimen.

In a first step, we aimed to analyze the reliability of the chosen blood-based biomarkers under identical exercise conditions using intraclass correlation coefficients [1]. In a second step, the aim was to retrospectively ascertain the feasibility whether it is possible to predict the outcomes of the first trial. In addition, the choice of a four-week interval was chosen to maintain relative consistency in the menstrual cycles of female subjects, thus minimizing its potential impact as a confounding factor.

### 4.7. Statistical Considerations

Our analytical approach is crucial for the results presented here; hence, the following implications should be acknowledged. K-means requires the optimal number of clusters. We determined the optimal number of *k* quantitatively and compared it with our a priori assumption. While there was agreement for most of the markers, IL-1RA and TBARS revealed additional subgroups (*k* = 3), comprising only 1-2 individuals with low silhouette values. In such cases, we decided to categorize these individuals as outliers and excluded them from further analysis. Since the classification results are highly influenced by the initial centroids' starting points, we decided to conduct 10,000 iterations with varying starting points and averaged them later.

Nonetheless, k-means enables the exploration of similarities in the overall trajectory of blood-based biomarkers from pre to 24 h post. This multivariate approach integrates all time points into the classification, reducing information loss compared to a single clustering time point, such as a threshold at 3 h post. A further important strength for our analytical approach lies in the experimental setting of the study. The high degree of standardization allowed us to conduct the classification across two distinct training sessions, enabling us to assess the temporal stability of the identified groups using the GAQ value.

### 4.8. Perspectives

Overall, the present data show that some molecular blood markers for the diagnosis of athletes' stress and recovery cycles exhibit a high degree of intraindividual stability and, therefore, are possible biomarkers. This is especially remarkable because studies often must deal with high standard deviations undermining successful analyses of group means. Based on the present analyses, IL-10, IL-6, IL-8, and CK seem to be suitable for the intraindividual diagnosis of exercise recovery cycles. In a practical sense, this could be additionally helpful in the prescription of regeneration treatments for “high” and “low” responding athletes by coaches or the medical staff. In addition to their stable regulation and thus high reliability, they also show a relation to subjective stress perception and functional muscular fatigue. It should be noted that for all markers, there are either groups of stronger and weaker responders or individuals with different basal levels. Such a finding must be included in the use of the markers and determined by preanalytics before the markers are used as biomarkers. At the same time, we would recommend analyzing the markers as a panel because they address different systems, such as metabolism, muscular integrity, and exercise-induced immune response. Accordingly, future studies should focus further factors influencing these markers and the practical use in athletic training.

## Figures and Tables

**Table 1:** Personal characteristics and anthropometric data.

Age (years)	25.61 ± 4.97
Height (cm)	176.71 ± 9.68
Weight (kg)	74.32 ± 14.64
BMI (kg/m^2^)	23.7 ± 3
HFmax (bpm)	194.22 ± 7.8
Max. lactate (mmol/L)	11.83 ± 2.38
Rel. VO_2_max (mL/min/kg)	45.9 ± 5.24

**Table 2:** Summary of statistics of the calculated regression trees and subsequent ranking of the best-performing biomarkers considering their prediction ability and GAQ.

Biomarker	Biomarker concentration at 24 h post		Difference in isometric quadriceps flexion (24 h post-pre)		Subjective feeling of exhaustion (BORG scale)		Summary	
	TD1	TD2	TD1	TD2	TD1	TD2	Σ RMSE	Ranking
	RMSE							
Cortisol	0.16	0.19	7.72	6.34	0.66	0.70	15.77	1
IL-1RA	0.32	0.41	9.19	6.69	1.16	1.15	18.92	2
IL-8	0.24	0.32	11.45	7.78	0.89	0.93	21.61	3
CK	0.35	0.47	11.58	8.51	1.10	1.19	23.2	4
TBARS	0.19	0.19	14.43	7.81	1.01	1.25	24.88	5
IL-15	0.39	0.35	17.43	13.35	1.11	1.46	34.09	6
LDH	0.35	0.34	18.19	13.31	1.66	1.40	35.25	7
IL-10	0.80	0.24	18.66	13.47	1.36	1.38	35.91	8
IL-6	0.26	0.41	20.79	14.10	1.90	1.81	39.27	9
CRP	0.63	0.75	23.47	15.81	1.87	2.00	44.53	10

## Data Availability

All data analyzed during this study are available upon reasonable request to the corresponding author.

## Abbreviations

ACTH: Adrenocorticotropic hormone
CK: Creatine kinase
CRH: Corticotropin-releasing hormone
CRP: C-reactive protein
GAQ: Group Assignment Quality score
HPA: Hypothalamic-pituitary-adrenal axis
HR: Heart rate
IAT: Individual anaerobic threshold
IL: Interleukin
LDH: Lactate dehydrogenase
MVC: Maximum voluntary contraction
RFT: Running field test
RMSE: Root mean squared errors
TBARS: Thiobarbituric acid reactive substances
TD: Testing day
TNF-*α*: Tumor necrosis factor-*α*
VO_2_max: Maximum oxygen consumption.

## Supplementary Materials

Supplementary Materials By calculating regression trees, we analyzed the potential of each marker to predict its reregulation as well as objective and subjective measures of performance and recovery. Since only the best-performing models are described in the manuscript, we attached a supplement file (S1), which includes the complete result tables with all calculated models for every output variable. Table S1.1 shows the results of the models predicting the biomarker concentration at 24 h post. Table S1.2 shows the results of the models predicting the difference in isometric quadriceps flexion at 24 h post. Table S1.3 shows the results of the models predicting the difference of subjective feeling of exhaustion at 24 h post.
