# Supplementary material for: Biomarkers in Endurance Exercise: Individualized Regulation and Predictive Value
Source: Transl Sports Med. 2023 Dec 14;2023:6614990. doi: 10.1155/2023/6614990 (PMC11022769; doi:10.1155/2023/6614990)
Supplement: Supplementary Materials — By calculating regression trees, we analyzed the potential of each marker to predict its reregulation as well as objective and subjective measures of performance and recovery. Since only the best-performing models are described in the manuscript, we attached a supplement file (S1), which includes the complete result tables with all calculated models for every output variable. Table S1.1 shows the results of the models predicting the biomarker concentration at 24 h post. Table S1.2 shows the results of the models predicting the difference in isometric quadriceps flexion at 24 h post. Table S1.3 shows the results of the models predicting the difference of subjective feeling of exhaustion at 24 h post. [file 6614990.f1.docx]

Supplement – S1

S1.1 Results of the models predicting the biomarker concentration at 24h post (RMSE).

| Biomarker | TD1 | | | TD2 | | |
| --- | --- | --- | --- | --- | --- | --- |
|  | Model 1 | Model 2 | Model 3 | Model 1 | Model 2 | Model 3 |
| Cortisol | 0.7 | 0.16 | 0.93 | 0.45 | 0.19 | 0.55 |
| IL-1RA | 0.64 | 0.32 | 0.64 | 0.67 | 0.41 | 0.71 |
| IL-8 | 0.67 | 0.24 | 0.74 | 0.67 | 0.32 | 0.73 |
| CK | 0.73 | 0.35 | 0.99 | 0.99 | 0.47 | 0.91 |
| TBARS | 0.67 | 0.19 | 0.58 | 0.49 | 0.19 | 0.57 |
| IL-15 | 1.06 | 0.39 | 1.01 | 0.65 | 0.35 | 0.8 |
| LDH | 1.28 | 0.35 | 1.11 | 1.13 | 0.34 | 1.02 |
| IL-10 | 0.99 | 0.8 | 1.05 | 0.31 | 0.24 | 0.33 |
| IL-6 | 0.44 | 0.26 | 0.47 | 0.49 | 0.41 | 0.72 |
| CRP | 0.96 | 0.63 | 0.95 | 0.87 | 0.75 | 1.13 |

S1.2 Results of the models predicting the difference in isometric quadriceps flexion at 24h post (RMSE).

| Biomarker | TD1 | | | TD2 | | |
| --- | --- | --- | --- | --- | --- | --- |
|  | Model 1 | Model 2 | Model 3 | Model 1 | Model 2 | Model 3 |
| Cortisol | 24.51 | 7.72 | 33.75 | 18.35 | 6.34 | 21.55 |
| IL-1RA | 25.08 | 9.19 | 30.61 | 17.33 | 6.69 | 21.15 |
| IL-8 | 25.26 | 11.45 | 31.03 | 18.46 | 7.78 | 24.93 |
| CK | 24.94 | 11.58 | 34.7 | 18.39 | 8.51 | 24.49 |
| TBARS | 24.84 | 14.43 | 40.21 | 17.21 | 7.81 | 19.76 |
| IL-15 | 25.41 | 17.43 | 32.17 | 18.49 | 13.35 | 21.8 |
| LDH | 27.81 | 18.19 | 31.74 | 19.13 | 13.31 | 25.02 |
| IL-10 | 24.9 | 18.66 | 32.08 | 18.38 | 13.47 | 24.33 |
| IL-6 | 25.57 | 20.79 | 30.3 | 18.39 | 14.1 | 23.48 |
| CRP | 25.58 | 23.47 | 27.25 | 18.48 | 15.81 | 19.19 |

S1.3 Results of the models predicting the difference of subjective feeling of exhaustion at 24h post (RMSE).

| Biomarker | TD1 | | | TD2 | | |
| --- | --- | --- | --- | --- | --- | --- |
|  | Model 1 | Model 2 | Model 3 | Model 1 | Model 2 | Model 3 |
| Cortisol | 2.09 | 0.66 | 3.09 | 2.18 | 0.7 | 2.94 |
| IL-1RA | 2.22 | 1.16 | 2.86 | 2.13 | 1.15 | 2.91 |
| IL-8 | 2.17 | 0.89 | 2.91 | 2.19 | 0.93 | 3.2 |
| CK | 2.16 | 1.1 | 2.39 | 2.19 | 1.19 | 2.73 |
| TBARS | 2.16 | 1.01 | 2.7 | 2.09 | 1.25 | 3.29 |
| IL-15 | 2.16 | 1.11 | 2.76 | 2.19 | 1.46 | 2.78 |
| LDH | 2.19 | 1.66 | 3.08 | 2.19 | 1.4 | 2.75 |
| IL-10 | 2.12 | 1.36 | 2.7 | 2.18 | 1.38 | 2.76 |
| IL-6 | 2.14 | 1.9 | 2.04 | 2.24 | 1.81 | 2.61 |
| CRP | 2.08 | 1.87 | 2.25 | 2.13 | 2 | 2.27 |
